# Molecular approach in the study of Alström syndrome: Analysis of ten Spanish families

**Published:** 2012-07-03

**Authors:** Teresa Piñeiro-Gallego, Marta Cortón, Carmen Ayuso, Montserrat Baiget, Diana Valverde

**Affiliations:** 1 Departmento de Bioquímica, Genética e Inmunología, Facultad de Biología, Universidad de Vigo, Vigo, Spain; 2 IIS- Fundación Jiménez Díaz, CIBERER Madrid, Spain; 3 Hospital de Sant Pau, Barcelona, Spain

## Abstract

**Purpose:**

To describe the clinical and genetic findings in 11 Spanish patients with confirmed (n=5) or suspected (n=6) Alström syndrome (AS).

**Methods:**

Patients underwent clinical evaluation, and were screened for variations in Alström syndrome 1 gene (*ALMS1*) using a genotyping microarray from Asper Ophthalmics and by direct sequencing of coding exons 8, 10, and 16 of *ALMS1*. Furthermore, we analyzed the presence of the A229T variant of retinitis pigmentosa GTPase regulator-interacting protein 1-like gene (*RPGRIP1L*) with direct sequencing of coding exon 6.

**Results:**

A great phenotypic variability was observed in our patients. Four mutations in *ALMS1*—two novel nonsense mutations in one family (p.Y1715X and p.S616X), one previously described mutation in homozygous state in another family (p.V3597Efs*4), and a likely pathogenic missense variation p.P1822L in a third family—were identified with direct sequencing. All patients were homozygous for 229A allele of *RPGRIP1L*, with the exception of a p.A229T heterozygous patient.

**Conclusions:**

Our findings expand the spectrum of *ALMS1* mutations causing Alström syndrome. The phenotypic differences between patients could be attributed to interactions with other genes inherited independently from the *ALMS1* gene or with environmental factors. A clear understanding of the phenotypic spectrum in AS will be important to unravel the molecular mechanisms underlying this syndrome.

## Introduction

Alström syndrome (AS, OMIM #203800) is a rare autosomal recessive disorder caused by mutations in Alström syndrome 1 gene (*ALMS1*; chromosome 2p13). With an estimated prevalence of 1.4 cases per 1,000,000 (Orphanet), to date only about 700 cases have been identified [1] since the condition was first described in 1959 [2]. Delayed diagnosis and misdiagnosis are common, making the estimation of the incidence of AS difficult [3,4]. AS is a multiorgan disorder characterized by cone-rod dystrophy, childhood obesity, progressive bilateral sensorineural hearing loss, insulin resistance, and type 2 diabetes mellitus (T2DM). Dilated cardiomyopathy with infantile or adult-onset occurs in more than 62% of patients [5]. Pulmonary involvement and hepatic, renal, and urological dysfunction are also frequently observed. Features reported in some but not all patients include acanthosis nigricans, male hypogonadism, hypothyroidism, mild to moderate developmental delay, and short stature.

*ALMS1* consists of 23 exons and encodes a novel protein of 4,169 amino acids (the National Center for Biotechnology Information [NCBI]) whose function remains unclear. However, the ALMS1 protein is widely expressed in most tissues affected [6,7], and localizes to basal bodies and centrosomes of ciliated cells, playing a possible role in intracellular trafficking [8–10]. A recent study [11] reported the implication of the ALMS1 protein in planar cell polarity signaling, indicating that the loss of cochlear hair cells causes the majority of the hearing loss in AS.

AS belongs to a growing class of human diseases with overlapping phenotypes, referred to as ciliopathies. These include disorders such as Bardet-Biedl syndrome (BBS), Senior-Loken syndrome (SLS), and Leber congenital amaurosis (LCA). The clinical similarity to these syndromes, particularly to BBS, and the onset delay of some of the clinical features in AS sometimes result in misdiagnosis [3,4]. Despite this similarity, we can distinguish patients with BBS from patients with AS because the latter develop significant sensorineural hearing loss and do not have polydactyly. LCA should be diagnosed in a young patient with vision loss, nystagmus, and severe retinal dysfunction without dilated cardiomyopathy, but diagnosis should be reconsidered if additional symptoms appear.

There is no specific curative treatment for AS, and the clinical purpose is to correct and relieve some of the symptoms, and to control the diabetes.

The aim of this work is to describe the clinical and genetic findings in 11 patients from ten families with confirmed or suspected AS.

## Methods

### Patients

Eleven Spanish patients were studied, five of them clinically diagnosed with AS and six suspected of having AS. The latter showed a major criterion and one or more minor criteria for AS diagnosis [12]. This study adhered to the tenets of the Declaration of Helsinki and was approved by an ethics committee. Informed consent was obtained from all study participants or their guardians after the nature of the procedures to be performed in this study were fully explained. The patients and their relatives were recruited through Fundación Jiménez Díaz (Madrid, Spain), Hospital de la Santa Creu i Sant Pau (Barcelona, Spain), and Hospital Vall d´Hebrón (Barcelona, Spain).

Control samples consisted of 50 unrelated Caucasian healthy individuals who did not have a personal or family history of retinal disease, and were provided by Complejo Hospitalario Universitario de Vigo.

The clinical diagnosis of Alström syndrome was based on the presence of major and minor clinical features that emerged throughout infancy, childhood, and adolescence [12]. Patient assessment included ophthalmologic, auditory, and general physical examinations. Diagnosis of retinal dystrophy was based on measurements of visual acuity and visual field tests, fundus examination, and electroretinogram (ERG). T2DM (fasting glucose), hearing loss (audiometric test), hepatic dysfunction, renal dysfunction, biochemical analysis, and karyotype analysis were assessed when possible.

### DNA extraction

Peripheral blood samples were collected from each member of the family in tubes containing EDTA. Genomic DNA was isolated with Flexi Gene DNA Kit (Qiagen, Hilden, Germany) following the instructions of the manufacturer for isolation of DNA from 4 to 14 ml whole blood.

### Genotyping microarray

All probands except patient 4 were screened for known mutations using the BBS/AS Asper Ophthalmics genotyping microarray (Asper Biotech, Tartu, Estonia). This microarray-based test screened for 313 previously known sequence changes in *ALMS1*, Bardet-Biedl Syndrome 1 gene (*BBS1*) to Bardet-Biedl Syndrome 10 gene (*BBS10*), and Bardet-Biedl Syndrome 12 gene (*BBS12*), Plant HomeoDomain-like finger protein 6 gene (*PHF6*; Borjeson-Forssman-Lehmann Syndrome), and Gs-protein alpha subunit 1 gene (*GNAS1*; Albright hereditary osteodystrophy).

Patients 3, 4, 5, and 6 were analyzed using the Asper Ophthalmics Leber congenital amaurosis (LCA) genotyping microarray (Asper Biotech) as previously described [13].

### DNA sequencing and mutation screening

Exons 8, 10, and 16 of *ALMS1* were amplified with PCR in an MJ Mini Gradient Thermal Cycler (Bio-Rad, Hercules, CA) with primers described by Collin et al. [6] (Table 1). We also amplified exon 6 of Retinitis pigmentosa GTPase regulator-interacting protein 1-like gene (*RPGRIP1L*) to evaluate the common variant p.A229T (c.685 G/A), a modifier of retinal degeneration in ciliopathies [14]. Primers used were previously described by Khanna et al. [14] (Table 1). PCR reactions were performed in a final volume of 25 μl containing 100 ng of genomic DNA, 0.4 μM of each primer, 0.8 mM dNTP Mix, 1.5 mM MgCl_2_, 1X PCR reaction buffer, and 1 unit of BIOTAQ™ DNA Polymerase (Bioline, London, UK). Program was as follows: initial denaturing at 94 °C for 5 min, followed by 35 cycles of 94 °C for 30 s, 52–66 °C for 35 s, and 72 °C for 30 s, followed by a final extension step at 72 °C for 10 min.

**Table 1:** Oligonucleotides for genomic amplification and sequencing of exon 8, 10, and 16 of *ALMS1* and exon 6 of *RPGRIP1L*.

**Gene**	**Exon**	**Forward Primer (5′-3′)**	**Reverse Primer (5′-3′)**	**Product size (bp)**	**Tm (°C)**
*ALMS1*	8.1	GCTTTTTAAAGGCTCAAAGCTG	GTATTCCCGTCTTCTGCTCCACT	646	52
	8.2	CAACTGGCATGTCAACTC	CTTCGGGTAGATGGCTGTC	666	56
	8.3	GTACCCACAGGACTTAGCA	ACTCCTGTTGATAGAAAATACTGG	731	58
	8.4	CTGACCAGAAGACTGTCCCAACAC	CAAGGCCTGCTGGTGGAAAAT	515	64
	8.5	CACACCAGCAGTACCGTCTAC	TCAGAGCCTCTTCAGTTGGATGATTA	527	66
	8.6	GAAAGTTTCACCTGTTCTTG	TGGTCCAGGAGCAGAAGAA	1021	59
	8.7	TGGCGCACCAACTATAACCTCTC	GCTGGTAGAAAATGACAGGCTTCC	491	64
	8.8	CTAAATAAAGAGGTTGTGAAAG	ATGTGAATAGAAAGAGGAAGTTA	375	53
	8.9	CAGGCCCTGCCAGACAGTGAG	CTGGCAACTCCTGCTGATGAAA	565	63
	8.1	GGTTCCTGGGCCTGCTGAC	TTGGGCTTTACTGTTTGAGAATAG	534	61
	8.11	TCACAAATAGAGAAGCCCAAGAT	ATGTGAAAAGGAACTAGGAAGAGC	375	54
	8.12	ATGTAACTGAAGATGTGCTGAAG	TTCTGCCTCCATCAAAAGTGTC	473	61
	8.13	AAGGATTTGCCAGATAGACAT	TCCTCTGTGAATGGCTGTCTGT	515	53
	8.14	ACTCTTAAGGAAATTCGGACAC	TTACAGATTTGGCTGCTTGATA	391	54
	8.15	CCTCTTCCACGGGTGTATCTAA	TGGCTAAGCTTCCTCAAAACA	710	56
	10.1	TGGTCTAATCTTAGCGTGGGT	ATGCATAGTAATTCACAAG	774	55
	10.2	ACACCATTTTCCCCTTCCT	CCGTGTGATTTCTCTGAGTGG	706	59
	10.3	TGTACTGGAGCATCTGTGGG	CAGTCAGCCCCAAATCACTG	860	63
	16.1	GCGAGGCTACTAAGCAACAAGGC	ACAGCCTGAGTTGGGTGAC	650	60
	16.2	CAGAAGGTCACCCCAGAG	CCATCTGGCAATGTGACTGC	835	63
*RPGRIP1L*	6	TTCACTGTGTGCAGAGGCACTT	TGCTCGACCTACTAACTGCTGTCA	447	62

PCR products of both genes were visualized on a 2% agarose gel containing 0.05% ethidium bromide and purified using NucleoSpin Extract II columns (Macherey-Nagel, Düren, Germany). The products were sequenced directly using the BigDye Terminator v1.1 Cycle Sequencing Kit (Life Technologies, Foster City, CA) in a 10 μl reaction. Sequencing products were precipitated, dried, and resolved in an ABI PRISM 3130 (Life Technologies) genetic analyzer. Sequence data were aligned to reference GenBank sequence NM_015120.4 and examined for variation. Detected mutations were confirmed in a second independent PCR reaction and identified in forward and reverse strands. Nucleotide and amino acid numbering of mutation sites began at the start codon ATG (Met) of the open reading frame based on GenBank NM_015120.4.

### Determination of pathogenicity

We used three sequence homology-based tools, Pmut [15], Polymorphism Phenotype (PolyPhen), and Sorting Intolerant From Tolerant (SIFT) [16], to predict the potential impact of an amino acid substitution [17].

## Results

We present five sporadic cases of AS, with an initial diagnosis of RP, except patient 5 who was diagnosed with congenital achromatopsia (ACHM) versus LCA. We also present six cases suspected of AS with no history of AS in their families. The clinical characteristics of the patients are described in Table 2 and Table 3.

**Table 2:** Summary of the clinical characteristics of patients with confirmed AS.

	**Patients (Family)**					
**Characteristics**	**# Cases**	**1 (RP1087)**	**2 (RP118)**	**3 (RP793)**	**4 (RP401)**	**5 (RP1232)**
Sex		F	M	F	F	F
Age		33	40	11	35	24
RD	5	+	+ (3y)	+ (8mo)	+ (16y)	+
Nystagmus	3	+ (5mo)		+ (9mo)		+ (1year)
Achromatopsia	2	+ (1wk)				+ (1year)
Photophobia	4	+ (9mo)		+	+ (1year)	+
Disiminished visual acuity	4	+ (2y)		+	+ (14y)	+
Night blindness	4	+ (2y)	+ (3y)		+ (1year)	+ (1year)
Diminished visual field	4	+ (2y)	+ (3y)		+ (14y)	+ (1year)
ERG/VEP	1/3	NR/-	NR/-	NR/NR		
T2DM	3	+ (25y)	+ (19y)	+ (9y)		
Obesity	4	+ (5mo)		+ (9mo)	+	+ (1year)
Sensorineural hearing loss	4	+ (21y)		+	+	+ (5y)
Acanthosis nigricans	3	+		+		+
MR	1				+	
Hypogonadism	1	+				
Renal defect	1					NP
Short stature	1			+		
Hypothyroidism	2			+		+ (11y)
DCM	1				+	
Hepatic defect	1			Steatohepatitis		
Orthopedic	2	Scoliosis		Scoliosis, Clinodactyly		
Other clinical symptoms	3			Ogival palate, Strabismus, Hyperopia	Ophthalmoplegia	Irregular menses, UBH, HA, HI, PCOS

F: Female; M: male; RD: Retinal dystrophy; ERG: electroretinogram; VEP: Visual evoked potentials; NR: No response; T2DM: Type 2 diabetes mellitus; NP: Nephronophthisis; MR mental retardation; DCM: Dilated cardiomyopathy; UBH: Unilateral breast hypoplasia; HA: Hyperandrogenism; HI: hyperinsulinemia; PCOS: Polycystic ovary syndrome. The age of onset of features is reported in parentheses.

**Table 3:** Summary of the clinical characteristics of patients with suspected AS.

	**Patients (Family)**					
**Characteristics**	**# Cases**	**6 (RP621)**	**7 (RP176)**	**8 (M304)**	**9 (GBB11)**	**10/11 (B66)**
Sex		M	F	M	F	F/F
Age		57	39	40	20	29/24
RD	6	+ (infancy)	+	+	+	+/+
Nystagmus	2		+		+ (3mo)	
Achromatopsia	1		+			
Disiminished visual acuity	3	+	+ (7y)		+	
Night blindness	3	+ (1year)	+ (4y)	+ (infancy)		
Diminished visual field	3	+ (1year)	+ (1year)	+ (11y)		
ERG/VEP	10/11	NR/-	Reduced/-	Normal/ Normal		
Obesity	6	+ (42y)	+	+ (15y)	+	+/+
Sensorineural hearing loss	1	+ (35y)				
Acanthosis nigricans	2			+	+	
MR	4			+	+	+/+
Hypogonadism	1			+		
Renal defect	1		Renal cyst			
Orthopedic	1				Small hands and feet	
Other clinical symptoms	3		Polyphagia	Hemeralopia (infancy) Dysplasia of the extremities, Facial dysmorphia	Hypoplasia of the dental enamel, Epileptic crisis (13y)	

F: Female; M: male; RD: Retinal dystrophy; ERG: electroretinogram; VEP: Visual evoked potentials; NR: No response; MR mental retardation. The age of onset of features is reported in parentheses.

**Figure 1:**
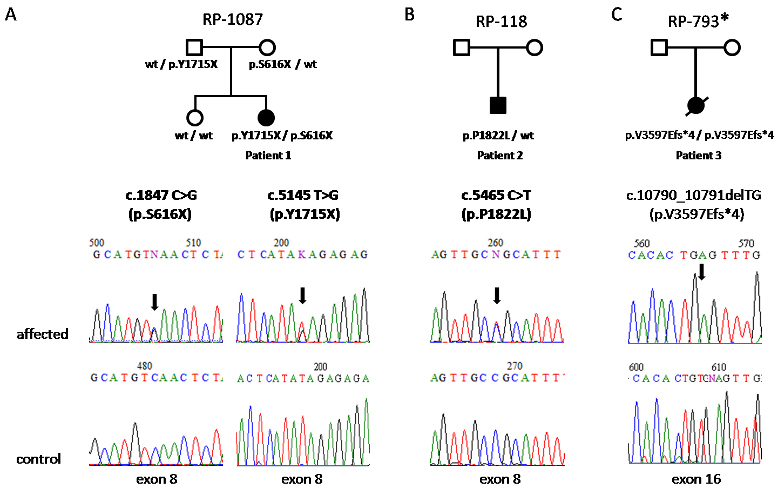
Pedigrees of families with Alström syndrome showing the segregation of the *ALMS1* mutant alleles found. Each pedigree is named with the family code. All the families were clinically diagnosed with Alström syndrome (AS). *mutation reported by Marshall et al. [18]. Novel mutations are in bold; wt denotes wild type.

No pathogenic mutations were found in the genes screened with Asper Ophthalmics microarrays used in this study (BBS/AS and LCA). Four mutations in *ALMS1* were identified with direct sequencing: two novel nonsense mutations in the family RP1087 (p.Y1715X and p.S616X), one previously described mutation (p.V3597Efs*4) in homozygous state (families RP1087 and RP793, respectively), and a likely pathogenic missense variation (p.P1822L) in heterozygous state (family RP118). Figure 1 shows the pedigrees of the AS families and the segregation of the *ALMS1* mutant alleles found.

Genomic DNA from patient 6 was unavailable for screening with Asper Ophthalmics microarrays, because the DNA sample was not large enough.

### Patients with Alström syndrome

#### Patient 1 (family RP1087)

She is the second of two children of healthy non-consanguineous parents. Mutation analysis of exons 8, 10, and 16 of *ALMS1* in this patient revealed two novel nonsense mutations in exon 8, c.1847 C>G (p.S616X) and c.5145 T>G (p.Y1715X). Her father was heterozygous for p.Y1715X mutation and her mother for p.S616X. The unaffected sister did not present these mutations. None of these mutations were present in 50 normal controls. This patient showed the genotype GG for *RPGRIP1L* c.685G>A, p.A229T.

#### Patient 2 (family RP118)

Patient 2 is the only son of healthy non-consanguineous parents.

A novel heterozygous amino acid change in *ALMS1*, c.5465 C>T leading to p.P1822L, was detected with direct sequencing. A simulation for functional changes by a structure homology-based method using the PolyPhen program resulted in classifying the P1822L change as probably damaging (Position-Specific Independent Counts, PSIC 2.724). According to this, P1822L is with high confidence supposed to affect protein function or structure. The web-based tool Pmut also predicted this change as pathological with an index of 0.6504 (indexes >0.5 signal pathological mutations) with a reliability of 3 [confidence index ranging from 0 (low) to 9 (high)]. However, this change is predicted as tolerated with a score of 0.07 by SIFT (substitutions with scores less than 0.05 are deleterious). P1822L was absent in 50 normal controls. This patient showed the genotype GG for *RPGRIP1L* c.685G>A, p.A229T.

#### Patient 3 (family RP793)

Patient 3 is the only daughter of healthy parents with no proven consanguinity; both are alive and with no apparent affect. The girl died at the age of 11.

In this patient, we identified a homozygous deletion in exon 16, c.10790_10791delTG, which causes a premature termination codon (p.V3597Efs*4) that predicts the truncation of the ALMS1 protein. This mutation has been previously described in another two Afro-Brazilian and Argentine families [18]. Parental genomic DNA was unavailable for allelic segregation analysis. This mutation was not present in 50 normal controls. This patient showed the genotype GG for *RPGRIP1L* c.685G>A, p.A229T.

#### Patient 4 (family RP401)

She is the second of two children of healthy non-consanguineous parents. She died, and information about her death is not available. Her sister died at 20 days of age due to cardiopathy.

The most common mitochondrial DNA mutations, m.A3243G in *MTTL1* [tRNALeu (UUR)] and m.C12258A in *MTTS2* [tRNASer (AGY)], and the most common mutation in *USH2A* (Usherin), c.2299delG, were excluded (Dr. Ayuso, personal communications). Mutation analysis of exons 8, 10, and 16 of *ALMS1* revealed no mutations. Genomic DNA from patient 4 was unavailable for mutation analysis of exon 6 of *RPGRIP1L*.

#### Patient 5 (family RP1232)

The affected patient was female, the first of three children of healthy consanguineous parents (fourth-degree cousins). Mutation analysis of exons 8, 10, and 16 of *ALMS1* revealed no mutations. This patient showed the genotype GG for *RPGRIP1L* c.685G>A, p.A229T.

### Patients suspected of having Alström syndrome

There were six patients from five families: patient 6 (family RP621), patient 7 (family RP176), patient 8 (family M304), patient 9 (family GBB11), and patients 10 and 11 (two siblings from family B66). Consanguinity was confirmed in family M304. Mutation analysis of exons 8, 10, and 16 of *ALMS1* revealed no mutations.

Four of the six patients showed the genotype GG for *RPGRIP1L* c.685G>A, p.A229T. Patient 9 showed the genotype GA for *RPGRIP1L* c.685G>A, p.A229T. Genomic DNA from patient 11 was unavailable for the mutation analysis.

## Discussion

To date, more than 100 different mutations have been described in *ALMS1*, including frameshift variations (deletions and insertions), nonsense mutations, one translocation [7], and a novel mutation caused by a recent Alu retrotransposon insertion in exon 16 of *ALMS1* [19].

Most of the mutations are present in exons 8, 10, and 16, being mutational hotspots, accounting for 25%, 27%, and 41% of the total mutational load in AS, respectively [20]. Exons 6, 12, 17, 18, and 19 also present mutations [18,21,22].

Screening for previously reported *ALMS1* mutations using the Asper Ophthalmics microarrays was negative. Nevertheless, this array has great potential as the first screening for cases of AS or BBS. In this way, in a previous work, we identified at least one likely causative mutation in 26.7% (36/135) of families of AS and in 40.5% (83/205) of BBS [23]. Furthermore, this genotyping microarray is more practical and cost-effective than direct sequencing of 23 coding exons of *ALMS1*. However, this microarray has several limitations such as novel mutations in the interrogated genes or variants in other genes not included in the array. As the number of new mutations in *ALMS1* increases, it is essential to update this array to improve its effectiveness.

In two patients with AS, two pathogenic alleles were detected with direct sequencing, and a potential pathogenic heterozygous variant in a third patient (patient 2). In patients suspected of having AS, we were unable to identify previously known pathogenic mutations or new variants. Our study confirms the usefulness of direct sequencing of the exons with a major contribution in *ALMS1* disease-causing variations (8, 10, and 16).

Patient 2 presented a novel amino acid change c.5465 C>T, in heterozygous state, leading to p.P1822L. This change seems to be a likely pathological variant based on i) its absence in 50 healthy unaffected controls; ii) its PolyPhen PSIC score of 2.724, which suggests that it may have a functional impact; and ii) its Pmut pathogenicity index of 0.6504. However, SIFT gives a contrary prediction; we have to take into account possible limitations of these software programs to validate the pathogenicity of variations identified during sequencing. The presence of one mutation together with clinical signs of AS in this patient could be interpreted as strong evidence for the pathogenicity of this novel variant.

The possible modifier role of *RPGRIP1L* [14] was assessed. All probands were genotyped for the variant allele A229T of this gene. All patients were homozygous for the 229A allele of *RPGRIP1L*, with the exception of a p.A229T heterozygous patient (patient 9). The 229T allele of *RPGRIP1L* has a possible role in the development of RP in patients with ciliopathy [14], but we were not able to document any influence on the phenotype of our patients.

The major clinical features usually observed in AS [12] were presented in our patients with confirmed AS. However, the overlap of AS features with those of BBS and other ciliopathies should be taken into account to make a correct diagnosis. Moreover, patients clinically diagnosed with AS harboring *BBS* mutations and patients with BBS with *ALMS1* mutations have been described [23]. For example, patient 3, with confirmed homozygous deletion p.V3597Efs*4, had clinodactyly, a sign not typical of AS. This sign has been described in BBS [24]; however, in patients with AS, digit anomalies have been rarely reported [5]. Patient 3 also had a high arched palate (Table 2), as observed in a cohort of 40 patients with BBS [24].

Patients suspected of having AS were initially diagnosed with other retinal-related pathologies, although the patients presented such classical AS features as retinal dystrophy, obesity, and sensorineural hearing loss and such variable symptoms as dental abnormalities, not always present in AS (Table 3). Four of the patients had delayed mental development, a feature not common in AS [12], and one had a renal cyst, a feature that has consistently been described in BBS. Although the patients presented characteristics related to BBS, the clinical overlap with AS observed in these subjects prompted us to include these patients in the study.

Most of the AS phenotypes are age-dependent; therefore, the first proposed diagnosis is sometimes wrong until additional symptoms are developed. This is the case of patients 3, 4, 5, and 6, where the presence of previously described mutations in the LCA genes was excluded by using Asper Ophthalmics LCA genotyping microarray. Patient 4 presented ophthalmoplegia, which is the most common manifestation in patients with mutations in mitochondrial DNA. Ophthalmoplegia along with dilated cardiomyopathy and RP suggest a mitochondrial disorder, Kearns-Sayre syndrome. However, mutation screening of mitochondrial DNA from a muscle biopsy of this patient was negative.

Diagnosis of AS is particularly difficult due to its low frequency and its complex phenotypes that are not present in infancy but develop throughout childhood and adolescence. Furthermore, the clinical similarity with other syndromes (BBS, SLS, LCA) makes diagnosis even more difficult, often resulting in misdiagnosis. In these cases, molecular genetic testing is indicated to confirm the diagnosis, which would allow evaluation of the progression of the disorder and awareness of other organ complications that could appear.

The difficulty of establishing a genotype-phenotype correlation is determined by the variable onset and outcome of the disease process. These phenotypic differences between patients could be attributed to interactions with other genes inherited independently from the *ALMS1* gene, as has been described in other ciliopathies with retinal degeneration [14], or could be attributed to environmental factors. The more molecular and clinical information we have, the easier a genotype-phenotype correlation could be established. A clear understanding of the phenotypic spectrum in AS, especially with the help of mouse models [9–11], may contribute to better insight in the future into the function of *ALMS1* at the molecular level.

In conclusion, the direct sequencing approach in those patients with a negative result in the preliminary screening with Asper Opthalmics microarray is a useful tool for molecular diagnosis of AS. Taking into account the mutations reported here, which extend the mutational spectrum in AS, could be useful for improving the array effectiveness in the diagnosis.
